# Alexithymia, Not Autism Spectrum Disorder, Predicts Perceived Attachment to Parents in School-Age Children

**DOI:** 10.3389/fpsyg.2020.00332

**Published:** 2020-03-17

**Authors:** Michele Giannotti, Simona de Falco, Paola Venuti

**Affiliations:** Department of Psychology and Cognitive Sciences, University of Trento, Trento, Italy

**Keywords:** autism spectrum disorder (ASD), attachment, alexithimia, emotion processing, school-age, parent-child relationship and ASD

## Abstract

Alexithymia is defined as a limited ability in the cognitive processing of emotions. Literature suggested its negative influence on interpersonal relationship, documenting elevated alexithymia in individuals with Autism Spectrum Disorder (ASD) compared to control groups. However, the study of alexithymia in school-age children with ASD remains largely unexplored as well as its effect on specific child socioemotional outcomes such as quality of attachment relationships. This study examines alexithymia and perceived attachment to parents in twenty-four children with ASD (without intellectual disability) and 24 typically developing (TD) children (mean age 10 years) using the self-reported Alexithymia Questionnaire for Children (AQC) and the Inventory of Parent and Peer Attachment (IPPA). Measures of family SES as well as child intelligence were collected. Data revealed that ASD children showed higher levels of Alexithymia compared to TD group. In addition, 21% of participants with ASD exceed alexithymia categorical cut-off. By contrast, no difference emerged in the perception of attachment to parents. Moreover, alexithymia, but not ASD status, was found to predictive of child perception of attachment to parents. We observed no significant effect of child age and verbal IQ. Our findings showed that alexithymia was more common in children with ASD, whereas attachment was similar between groups. Difficulties in identifying and describing one’s own feelings may hinder the construction of a positive representation of parent-child attachment relationship regardless of child clinical status. Thus, alexithymia seems to play a key role on the way school-age children with and without ASD perceive their relationship with their parents.

## Introduction

Autism spectrum disorder (ASD) is a neurodevelopmental condition characterized by sociocommunicational impairments and restricted and repetitive patterns of behaviors and interests (American Psychiatric Association [APA], 2013). Empirical studies have widely documented the presence of significant difficulties in the domain of emotion processing and regulation in ASD (Silani et al., 2008). In particular, during the last two decades, the construct of alexithymia has received greater attention in the arena of emotion processing in ASD (Kinnaird et al., 2019). Namely, the concept of alexithymia refers to individual difficulties in identifying, describing, and distinguishing one’s own feelings, which are often accompanied by an externally oriented thinking instead of a focus on internal experience (Sifneos, 1973). Previous studies have consistently shown elevated alexithymia in individuals with ASD compared to control groups, regardless of the level of intellectual abilities (Hill et al., 2004; Lombardo et al., 2007; Griffin et al., 2016), reporting higher rates of adults with ASD above the clinical level (Bird and Cook, 2013). Nevertheless, only a few studies investigated alexithymia in children and adolescents showing similar findings (Griffin et al., 2016; Milosavljevic et al., 2016).

Interestingly, a series of studies found that alexithymia and not ASD status underpinned the specific emotion processing difficulties reported in this clinical population (Bird et al., 2010; Heaton et al., 2012). This area of research did not attribute emotion-processing and interoception difficulties to ASD core symptoms, but suggested instead a predictive effect of alexithymia (Bird and Cook, 2013; Shah et al., 2016). In addition, several studies underlined a considerable overlap between ASD and alexithymia neurobiological and anatomical correlates (van der Velde et al., 2013), including altered activation of specific brain areas such as amygdala and anterior cingulate cortex (Bernhardt et al., 2013; Caria and de Falco, 2015). Moreover, alexithymia as well as ASD are associated with impaired mentalizing abilities (Moriguchi et al., 2006), possibly in light of the reduced integration between physiological states interoception and emotional consciousness (Gaigg et al., 2018). For these reasons, scholars suggest considering alexithymia as a significant predictor of developmental outcomes of individuals with ASD and a key concept to identifying cognitive profiles of specific subgroups within the ASD heterogeneity (Lai et al., 2013; Fietz et al., 2018). Despite its clinical relevance, prior research on ASD focused almost exclusively on adulthood – hence, the investigation of alexithymia in childhood remains largely unexplored. To date, only one study (Griffin et al., 2016) has been conducted on young children, confirming a higher level of alexithymia for the ASD group using both self- and parent-reports.

### Attachment and Alexithymia in ASD

Among the several factors associated with alexithymia, quality of attachment to parental figures constitutes a crucial predictor of children’s healthy psychological development (Carlson and Sroufe, 1995), showing a significant contribution to several indicators of socioemotional adjustment and adaptation. In fact, developmental research has documented a robust relationship between insecure attachment to caregiver and emotion processing difficulties (Laible, 2007; Thompson and Meyer, 2007; Brumariu et al., 2012). However, the association between attachment and alexithymia has been mostly investigated in adulthood (Picardi et al., 2005). In fact, studies on children and adolescents focused on this link are still scarce, particularly on clinical samples (Oskis et al., 2013; Koelen et al., 2015). Therefore, there is a substantial lack of research concerning the association between alexithymia and attachment in children with ASD. A recent study (Costa et al., 2019) found that alexithymia predicts reduced parent–child interaction more than ASD status, suggesting the need to consider its impact on quality of relationship in future research. In fact, the study did not include a specific measure of attachment relationship to parents, which represents a core feature of child socioemotional development.

In this regard, literature on attachment showed no differences between ASD and their typical counterparts in the perception of quality of attachment to parents (Bauminger et al., 2010: Chandler and Dissanayake, 2014; Sivaratnam et al., 2018). Nonetheless, the study of attachment in ASD during middle childhood remains poorly investigated and further replications are needed to clarify the absence of significant differences with typical controls. Additionally, understanding the mechanism underlying the perception of attachment security to parents in children with ASD may elucidate the association among key aspects of socioemotional development in this clinical population, explaining which child features may contribute to positive explicit representations of trustworthy, sensitive, and available parents. Given that neither ASD status nor symptoms severity showed a significant influence on the perception of attachment security in ASD, it could be interesting to consider the role of alexithymia in predicting this socioaffective domain. According to a bidirectional perspective, child characteristics may influence the quality of parent – child interaction (Costa et al., 2019), altering parental attitudes and caregiving behaviors. Therefore, specific subclinical phenomenon such as impairments in identifying and describing one’s own feelings as well as difficulty in distinguishing emotion from bodily sensations (Silani et al., 2008) can affect the way in which children perceive their attachment relationship with their parents.

The present study aimed at investigating alexithymia in school-age children with ASD (without intellectual disability) by examining its predictive role on child perception of attachment security to parents. Firstly, we explored potential differences between groups for both attachment and alexithymia. To this purpose, we also estimated the percentage of children above the normative cut-off of alexithymia. Next, the predictive effect of child features, including alexithymia, were tested with the aim to identify which mechanisms intervene in shaping the perception of attachment security to parents in ASD. We primarily hypothesized to find higher levels and rates of alexithymia in children with ASD compared to the controls, according to previous studies on this topic (Kinnaird et al., 2019). No differences were expected with respect to the perception of attachment security to parents as highlighted by prior research (Teague et al., 2017). In regard to our second aim, we expect to find a significant link between alexithymia and attachment according to literature on neurotypical population.

## Method

### Procedure

Participants with ASD diagnosis were recruited through two different clinical centers for children with neurodevelopmental disorders. Clinicians informed parents of children with a certified ASD diagnosis who meet inclusion criteria about the possibility to be involved in this study. We used snowball sampling and specific advertisements in the University area to recruit children with typical development. In response to the expression of interest of the parents, we invited the families to the clinical centers to participate in this study. After reading a detailed informative of the study, parents signed the informed consent, including the form concerning protection of personal data. This procedure has been accomplished according to the EU General Data Protection Regulation (GDPR) no. 2016/679. This study was given ethical approval by the Ethic Committee on Experiments involving human beings of the University of Trento. The administration of the questionnaires was conducted by an experimenter in a quiet room of the centers involved in the study. Mothers of children with TD were asked to complete the Social Responsiveness Scale 2 (Constantino and Gruber, 2005) in order to screen participants of the control group for sociocommunicational impairment. Similarly, the Autism Diagnostic Observation Schedule (ADOS, Module 3; Lord et al., 2015) was used to confirm diagnosis in children of the ASD group. We also collected measures of verbal and non-verbal intelligence and family socioeconomic status in both groups. Moreover, children completed two self-report questionnaires for the assessment of alexithymia and quality of attachment to parents.

### Participants

Out of the 52 contacted families, 4 refused to participate (2 of ASD and 2 of the TD group) – hence, our final sample included 24 children with ASD (without intellectual disability) and 24 children with typical development (TD). Children mean age is 126.4 months (*SD* = 16.45) for the clinical group and 115.88 months (*SD* = 25.14) for the normative group. The majority of the participants involved in this study were males (75% of the total sample and 62.5% of TD), particularly in the ASD group (*n* = 19; 87.5%). Family Socioeconomic Status (SES; Hollingshead, 1975) is similar for both groups ranging from medium to high. All the children of the clinical group had a certified clinical diagnosis of ASD without intellectual disability (IQ greater than 70), based on clinical judgment according to the *Diagnostic Statistical Manual of Mental Disorders 5th Edition* (DSM V; American Psychiatric Association [APA], 2013). Children with intellectual disability, severe impairment of cognitive functioning, co-occurring psychiatric disorders, and deficits in expressive and receptive language were excluded from the study. For the TD group, we did not include children with a history of psychiatric disorder.

### Measures

#### Verbal IQ

*Wechsler Scale of Intelligence for Children* (WISC-IV; Wechsler, 2003) is the most widely used standardized tool in the field of developmental psychological assessment. According to the methodology and aims of this research, we used two WISC core subscales (Similarities and Vocabulary) to generate an index of child verbal intelligence. Similarities provide an estimation of child verbal abstract reasoning. This core subtest also involves language development, lexical knowledge, auditory comprehension, memory, and the ability to discriminate between essential and non-essential features. Vocabulary (VOC) offers a measure of child lexical knowledge and formation of verbal concepts.

#### Non-verbal IQ

*Raven Colored Progressive Matrices* (CPM; Raven et al., 1962) is a widely recognized individual non-verbal assessment of children intelligence based on figural materials. Specifically, this tool evaluates non-verbal perceptual and inductive reasoning in children from 3 to 11 years old independently from the culture or cognitive impairment. It consists of three series of 12 items developed to measure the main characteristic processes of this age group. A general weighted score was calculated by adding the child’s correct answers.

#### Attachment to Parents

*Inventory of Parent and Peer Attachment* (IPPA; Armsden and Greenberg, 1987) is a self-reported measure aimed to assess how children and adolescents perceive their parents and close friends as a source of psychological security. In this study, we used the version related to the relationship with parents only. It comprises 28 items rated on a five-point Likert-type scale from 1 = “almost never or never true” to “almost always or always true,” which generates three subscales (Trust, Communication, and Alienation) and a total score. Higher scores indicate a positive perception of the attachment relationship. This questionnaire showed adequate psychometric properties (Jewell et al., 2019), and it has been used to investigate the perception of attachment security in children with ASD (without intellectual disability) during middle childhood (Teague et al., 2017).

#### Alexithymia

*Alexithymia Questionnaire for Children* (AQC; Rieffe et al., 2006) is a self-reported measure to assess alexithymia in young children. It is adapted from the well-validated measure used for the assessment in adulthood (TAS-20; Bagby et al., 1994) to be developmentally appropriate preserving similar structure and content. The AQC is composed of 20 items rated on a three-point Likert-type scale (ranging from 0 = not true to 2 = often true) representing three core factors: (a) Difficulty Identifying Feelings (DIF; seven items): (b) Difficulty Describing Feelings (DDF; five items); and (c) Externally Oriented Thinking (EOT; eight items). An example of an AQC item is “I can easily say how I feel inside.” Five items of the scale were formulated positively, for example, “It is important to understand how you feel inside” and thus the scoring was reversed.

In this study, we used the Italian version of the questionnaire (Di Trani et al., 2009, 2018). Higher scores correspond to the elevated presence of this factor. The validity and reliability of this measure were confirmed by empirical data (Rieffe et al., 2006; Di Trani et al., 2009, 2018), except for EOT, which showed low reliability. In our sample, AQC internal consistency was good for the total score (α = 0.678), DIF (α = 0.718), and DDF (α = 623) and poor for EOT (α = 0.035). This measure has already been used in children with ASD and other neurodevelopmental disorders (Donfrancesco et al., 2013; Griffin et al., 2016).

### Data Analysis

The statistical analysis of the data was carried out using the statistical package SPSS (22.0 for Windows). As a preliminary analysis, we checked for the normality of the distribution, outliers, and linearity and we tested bivariate correlations among alexithymia and attachment scores. A one-way multivariate analysis of variance (MANOVA) was used to test group differences on the control variable in order to detect potential covariates. Similarly, with chi-squared test, we assessed potential differences between groups regarding gender distribution. In addition, we transformed IPPA and AQC total scores into *z* scores in order to standardize both variables of interest. With respect to the first aim of the study, we performed a Mann–Whitney test to compare the two study groups on attachment total scores. For group differences on alexithymia, we performed a Student’s *t*-test for the total score and a one-way MANOVA for the three subscales of the questionnaire. One outlier (1 TD) was removed from the statistical analysis. Using a categorical cut-off score based on the normative values of AQC Italian validation study (Di Trani et al., 2018), we calculated rates of children at risk of alexithymia. Specifically, we determined the threshold parameters using the normative means + 1 SD clustered by two age groups (8–10 and 11–14 years old) and child gender. Differences between groups on this categorical variable were tested using the Fisher exact test. Next, we used a hierarchical linear regression to test which child variables contributed significantly in predicting child attachment to parent scores. Specifically, the first step of the regression model included child age, verbal IQ, and ASD status (presence/absence). In the second step of analysis, we added child self-reported alexithymia score to test its independent predictive effect on the overall IPPA model. A third step was included to test whether the effect of the alexithymia on attachment is moderated by the ASD status.

## Results

The AQC scores were normally distributed in both ASD and control group as opposed to IPPA total scores. Children with ASD did not differ from the TD group with respect to the control variables such as child age, verbal and non-verbal ability, and family SES (see Table 1). Similarly, no difference was found in the gender distribution between groups (Fisher exact test, *p* = 0.09). Correlational analysis is shown in Table 2. By addressing the first study aim, there was a significant difference between the two groups on AQC total score [*t*_(__45__)_ = −2.36, *p* = 0.022, Partial eta squared = 0.111] with children of the ASD group showing a higher level of alexithymia. The MANOVA was significant for the group effect on the AQC subscales [Wilk’s Lambda = 0.821, *F*(3,43) = 3.12, *p* = 0.035]. Among the univariate tests, only the DDF subscale was significant, *F*(1,45) = 5.65, *p* = 0.022, whereas a prominent trend that approached significance was found for the EOT subscale, *F*(1,45) = 3.76, *p* = 0.059.

**TABLE 1:** Descriptive statistics of the study variable for the ASD and TD group.

	**ASD**		**TD**		**Group differences**	
	***M***	***SD***	***M***	***SD***	***p***	***Eta***
Age (months)	126.43	16.45	117.52	24.32	0.185	0.045
Verbal IQ	22.45	4.00	22.91	4.94	0.722	0.003
Perceptual reasoning^a^	0.52	80	0.67	0.78	0.590	0.008
Family SES	43.52	13.79	45.39	15.23	0.607	0.007
Alexithymia total score	39.50	5.75	36.09	3.90	0.022*	0.111
Difficulty identifying feelings	13.12	3.55	12.13	2.41	0.022*	0.112
Difficulty describing feelings	10.16	2.38	8.65	1.94	0.270	0.027
Externally oriented thinking	16.20	2.62	14.91	1.88	0.059	0.077
Attachment to parents	61.19	8.442	66.43	13.75	0.090	0.064

ASD, autism spectrum disorder; TD, typical development; IQ, intelligence quotient; SES, socioeconomic status. ^*a*^*z* scores; *p < 0.05.

**TABLE 2:** Spearman correlations among perceived attachment to parents and alexithymia total score and subscales.

**Variable**	**1**	**2**	**3**	**4**	**5**
1. Alexithymia total score	–				
2. Difficulty identifying feelings	0.810***	–			
3. Difficulty describing feelings	0.745***	−0.501***	–		
4. Externally oriented thinking	0.483**	0.139	0.124	–	
5. Attachment to parents	−0.552***	−0.402**	−0.398**	−0.321*	–

*p < 0.05; **p < 0.01; ***p < 0.001.

In addition, we found higher rates of alexithymia in the ASD group; specifically 20.8% of children with ASD were above the cut-off compared to 8.3% of the typical development group. However, only a marginal trend toward significance emerged from the analysis (Fisher exact test, *p* = 0.091). The same analysis was conducted without removing the outlier showing similar results. In this case, though no statistical differences emerged on AQC total score, we found a substantial trend toward significance [*t*_(__46__)_ = −1.90, *p* = 0.063]. No significant differences were found between children with ASD and the control group on IPPA total scores (*Z* = 1.69, *p* = 0.090, Partial eta squared = 0.064). To ascertain the effect of child features and the contribution of alexithymia, a hierarchical linear regression including two separate steps was carried out (see Table 3). The first step that included child age, verbal IQ, and ASD status was not statistically significant [*F*_(__3_,_42__)_ = 2.44, *p* = 0.078]. Nevertheless, child age was positively associated with attachment to parents (β = 0.349), whereas the contribution of verbal IQ and ASD status was not statistically significant. By entering AQC total score as an independent predictor in the second step of the linear regression, the overall model was significant [*F*_(__4_,_42__)_ = 3.54, *p* = 0.015], explaining 19.5% of the variance. The *p*-value associated with adjusted R squared change for the second step is also statistically significant [*F*_(__1_,_38__)_ = 5.93, *p* = 0.020]. Specifically, the data revealed that alexithymia was the significant independent negative regressor (β = −0.361), whereas no effect was found for child age, verbal IQ, and ASD status. Similarly, alexithymia was still significant in the third step of the regression (β = −1.10; *p* = 0.040), whereas no interaction effect with ASD status was observed.

**TABLE 3:** Hierarchical regression analysis of perceived attachment to parents by child age, verbal IQ, ASD status, and self-reported alexithymia.

**Predictors**	**Attachment to parents**				
	**β**	***SE***	***p***	**Δ*R*^2^**	***p ch***
**Step 1**			0.078	0.158	
Age	0.349	0.084	0.030*		
Verbal IQ	0.122	0.398	0.424		
ASD status	–0.285	3.51	0.066		
**Step 2**			0.015*	0.114	0.020*
Age	0.255	0.082	0.099		
Verbal IQ	0.110	0.375	0.446		
ASD status	–0.172	3.48	0.257		
Alexithymia	–0.361	0.350	0.020*		
**Step 3**			0.13*	0.041	0.144
Age	0.250	0.080	0.100		
Verbal IQ	0.071	0.375	0.620		
ASD status	–1.85	26.58	0.111		
Alexithymia	–1.10	1.23	0.040*		
Alexithymia * ASD status	2.02	0.713	0.144		

ASD, autism spectrum disorder; TD, typical development, IQ, intelligence quotient; SES, socioeconomic status; *p < 0.05.

## Discussion

The current study examined alexithymia in school-age children with ASD (without intellectual disability), exploring its influence on the perception of attachment security to parents. To this aim, we tested the hypothesis that alexithymia may contribute to a negative view of the quality of the relationship with parents from the child’s perspective. To date, there are no studies that have investigated this specific link in children with atypical neurodevelopmental condition, including ASD.

Firstly, we found that children with ASD and TD showed no significant differences in the perception of attachment security to parents. Although a limited number of studies have been conducted on this topic, our results are consistent with previous findings. According to earlier meta-analytic findings, recent empirical evidence revealed that school-age children with ASD (without intellectual disability) reported similar levels of security in the relationship with their parents to those found in typically developing children (Teague et al., 2017). However, considering the negative impact of ASD sociocommunicational difficulties and emotional reactivity on quality of attachment bond and parent – child interaction, these findings raised questions about which mechanism may explain child-positive perception of the attachment relationship.

Secondly, in line with the only study available (Griffin et al., 2016), our results showed that school-age children with ASD reported higher levels of alexithymia compared to their typical counterpart.

Specifically, children with ASD reported more difficulties in describing their feelings and inner states.

Moreover, we found that alexithymia is more common in ASD, also in school-age, with approximately one in five reporting scores of alexithymia above the cut-off. Our finding confirms the difficulties of children with ASD in cognitive processing of their own emotions, documented by previous research on adolescent and adulthood (Bird and Cook, 2013; Milosavljevic et al., 2016). As expected, we did not find a large effect on this group difference given that the children’s self-report may be less reliable compared to other ratings provided by child informants. In fact, the limited abilities of individuals with ASD with respect to self-referential cognition (Lombardo et al., 2007) may undermine the accuracy of self-reported measurement. It is essential to consider other aspects associated to ASD phenotype that are strictly interrelated to alexithymia (Fitzgerald and Bellgrove, 2006) such as impairment in mentalizing and self-reflection, less coherent representations of emotional experience (Losh and Capps, 2006), absence of emotional vocalization (Heaton et al., 2012), behavioral rigidity, and impaired inhibitory control (Mosconi et al., 2009; D’Cruz et al., 2013). Thus, even if we checked for linguistic abilities, elevated levels and rates of alexithymia in children with ASD may be explained at least to some extent by these specific disorders of cognitive, emotional, and behavioral functioning.

According to the second aim of this study, we found a significant link between alexithymia and perception of attachment security in children with and without ASD. In particular, among child characteristics, alexithymia level and not ASD diagnosis predicts the extent to which children perceive their relationship with their parents as a source of security in middle childhood. It is conceivable that a specific deficit in identifying and describing one’s own feelings may hinder the construction of a positive representation of parent – child attachment relationship regardless of child clinical status. With respect to ASD, despite a growing area of research linking alexithymia and children psychological outcomes (Brewer et al., 2015; Morie et al., 2019), this is the first investigation documenting the significant impact of alexithymia on the perception of attachment security to parents. An ongoing debate in ASD is whether the occurrence of alexithymia affects social motivation, influencing attitudes and behaviors at an interpersonal level (Pastore et al., 2019). Studies reported that alexithymia in children with ASD was associated with less expressive coherence (Costa et al., 2017), empathy, and perspective taking (Lartseva et al., 2015) as well as lower enjoyment of prosocial interactions (Gebauer et al., 2014). Moreover, as highlighted by Costa et al. (2019), alexithymia in children (more than ASD status) can negatively affect parent–child relationships, explaining the reduced amount of dyadic exchanges. The mismatch between arousal activation and subjective experience of feelings (Gaigg et al., 2018) may also contribute to the formation of less coherent child representation influencing how information is encoded and processed. Therefore, the possibility to develop unbalanced representations of the attachment figures may significantly increase given the potential negative consequences of alexithymia on different levels of emotion processing and relational exchanges. Furthermore, giving a coherent meaning to their own interpersonal experiences with parental figures may be more complex for the limited personal resources in emotional self-understanding, regulation, and expression. In fact, children with ASD and alexithymia may also exhibit difficulties in interpreting and responding to emotion in others (Poquérusse et al., 2018) as in the case of child caregivers. Impaired mentalizing associated with low self-memory in ASD (Lombardo et al., 2007) and difficulties with episodic autobiographical memories (Lind, 2010) constitute additional risk factors for the construction of coherent explicit representations based on past relational experiences. Following this direction, our findings extend literature on the influence of alexithymia on socioemotional development by considering a child’s self-perspective on attachment to parents during school age.

In sum, our findings showed higher levels of alexithymia compared to the control group, whereas the perception of attachment security was similar between groups. Notably, alexithymia, not autism, was found to be the only significant predictor of child attachment to parents. Thus, alexithymia seems to play a key role on the way children with ASD perceive their relationship with their mothers and fathers. In this regard, several aspects associated with alexithymia such as impaired emotion processing, neurophysiological atypical processes, reduced mentalizing, and low self-memory may hamper parent – child relationship and consequently child-explicit representations.

Lastly, some limitations of this study need to be acknowledged. We do not include a measure of alexithymia rated by child informants as suggested by prior research (Griffin et al., 2016). Thus, a comparison between two different sources of information on alexithymia scores was not possible.

Additionally, a small sample size, a cross-sectional design, and the lack of a continuous measure of ASD symptoms severity represent other specific drawbacks of this study. Future research can expand these findings including a measure of child-implicit attachment representations. In general, our results confirmed that alexithymia could be useful in subgrouping and identifying specific cognitive profiles within the autism spectrum condition. We also suggest alexithymia as a potential covariate in the comparative study on ASD. Another possible indication is to assess alexithymia in parents to examine whether there are direct associations with child outcomes. In terms of clinical implications, we recommend assessing alexithymia adequately in school-age children with ASD in order to acquire specific information on their emotional functioning. Children with ASD and co-occurring higher level of alexithymia may benefit from interventions that combine evidence-based treatment for sociocommunicational difficulties and specific strategies aimed at enhancing the cognitive processing of their own emotions. In conclusion, our findings confirmed the importance to target children alexithymia in ASD, considering its clinical significance not only on emotion processing but also on other significant domains of socioemotional development, as is the case of attachment to parental figures. Interventions involving mothers and fathers aimed at boosting the quality of relational exchanges and child emotional capacities should evaluate and address alexithymia given its prominent contribution to child adjustment in ASD during middle childhood.

## Data Availability Statement

The raw data supporting the conclusions of this article will be made available by the authors, without undue reservation, to any qualified researcher.

## Ethics Statement

The studies involving human participants were reviewed and approved by the Human Research Ethics Committee of the University of Trento. Written informed consent to participate in this study was provided by the participants’ legal guardian/next of kin.

## Author Contributions

MG, SF, and PV contributed to the design and implementation of the research. MG collected and analyzed the data. All authors discussed the results and commented on the manuscript.

## Conflict of Interest

The authors declare that the research was conducted in the absence of any commercial or financial relationships that could be construed as a potential conflict of interest.
